# High prevalence of chronic musculoskeletal complaints among women in a Norwegian general population: the Tromsø study

**DOI:** 10.1186/1756-0500-7-506

**Published:** 2014-08-08

**Authors:** Ole Fredrik Andorsen, Luai A Ahmed, Nina Emaus, Elise Klouman

**Affiliations:** Department of Community Medicine, Faculty of Health Sciences, University of Tromsø, 9037 Tromsø, Norway; Department of Health and Care Sciences, Faculty of Health Sciences, University of Tromsø, 9037 Tromsø, Norway

**Keywords:** Musculoskeletal complaints, Population-based, Cross-sectional, Prevalence, Norway, Self-perceived health, Gender

## Abstract

**Background:**

The aims of this study were to estimate the prevalence and severity of MSCs in the adult general population of Northern Norway, and to study associations between MSCs and various demographic and lifestyle variables.

**Methods:**

Data from the Tromsø 6 survey (2007–2008) of the population-based Tromsø Study were used (12,984 participants, 65.7% participation rate). We included 8,439 participants aged 30–79 years in the analyses. Associations between demographic and lifestyle variables and chronic MSCs (i.e., those lasting for at least 3 consecutive months, hereafter referred to as simply MSCs) was examined using logistic regression analysis.

**Results:**

The total age-adjusted prevalence of both mild and severe MSCs was 63.4% and 52.9% in women and men, respectively. In women, the age-adjusted prevalence was 44.0% and 19.4% for mild and severe MSCs, respectively; the corresponding values in men were 40.8% and 12.1%. The highest prevalence was found in the neck/shoulder region (34.2% and 8.9% for mild and severe MSCs, respectively). The prevalence of MSCs in ≥5 body regions was three times higher in women than in men (14.9% vs 5.6%). Current smoking was significantly associated with MSCs (odds ratio [OR]: 1.41, 95% confidence interval [CI]: 1.22-1.62), but showed a stronger effect in women (OR: 1.60, 95% CI: 1.30-1.96) than in men (OR: 1.25, 95% CI: 1.02-1.52). Self-perceived poor health was strongly associated with MSC (OR: 3.73, 95% CI: 3.27-4.24). Moderate vs low level of physical activity was associated with MSCs only in women (OR: 1.37, 95% CI: 1.12-1.67). Other demographic and lifestyle variables associated with MSCs were age (OR: 1.04, 95% CI: 1.01-1.06), body mass index (BMI) >30 kg/m^2^ (OR: 1.42, 95% CI: 1.23-1.66), low education level (OR: 1.78, 95% CI: 1.53-2.08) and former smoking (OR: 1.21, 95% CI: 1.09-1.35). Marital status, BMI <18.5 kg/m^2^, high and very high level of physical activity was not associated with MSCs.

**Conclusion:**

Chronic MSCs are highly prevalent in this Northern Norwegian population, and are strongly related to self-perceived poor health. Women have a higher burden of MSCs than men. Most demographic and lifestyle variables associated with MSCs showed stronger associations in women than in men.

## Background

Musculoskeletal complaints (MSCs) are a major and costly health problem in Norway. MSCs are often the object of visits to general practitioners; they are a common reason for sickness absence from work, and represent a heavy burden on the disability pension fund in Norway [1–3]. The reported prevalence of MSCs in different populations varies from 17.1% to 78.6% [4–15]. Differences in case definition, response rates and cohorts may explain these large variations [16]. Many studies reported gender differences in their prevalence estimates [4, 6, 8, 10, 13, 17], while others did not [5, 11, 14]. Some authors have reported that the prevalence of MSCs increases steadily with age [11, 13, 17], while others have described a peak in prevalence at younger ages [4–6, 10, 12, 14, 15].

Associations between MSCs and several negative health determinants, such as smoking, overweight, education level, and low level of physical activity, have been suggested, but are not consistent across studies [4, 5, 10, 11, 13, 18, 19]. To develop more effective preventive strategies, we need to understand more about the gender- and age-specific distribution of MSCs, as well as the main factors with which they are associated. Until now, only prevalence data on neck/shoulder pain and headache have been reported from Northern Norway [20]. Tromsø is the largest city in Northern Norway. It is situated ≈ 400 km north of the Arctic Circle, and has approximately 70,000 inhabitants. The population of Tromsø is relatively well educated, and the city has its own university. The physical living conditions are dominated by dramatic changes in sunlight, with 2 months of midnight sun and 2 months of polar night.

The aims of this study were to estimate the prevalence and severity of MSCs by age and gender in a large adult population in Northern Norway, and to study the association between MSCs and various demographic and lifestyle variables.

## Methods

### The Tromsø study

The Tromsø Study is a longitudinal, population-based, multi-purposed health study carried out in the municipality of Tromsø, Northern Norway. The study consists of six repeated surveys and medical examinations administered between 1974 (the Tromsø 1 survey) and 2007–2008 (the Tromsø 6 survey). Each survey was conducted in two phases, with the most basic examination conducted at the first visit, and more extensive examinations done at the second visit.

The Regional Committee of Research Ethics and the Norwegian Data Inspectorate recommended that the Tromsø 6 survey be carried out, and written informed consent was obtained from all participants.

### Participants

All residents of Tromsø ≥25 years of age were invited to participate in the Tromsø 4 survey (1994–1995), and a large subgroup of these (n = 7,965) also attended the second visit of the Tromsø 4 survey. Those who still resided in Tromsø in September 2007 were invited to the Tromsø 6 survey, along with some whole birth cohorts and random samples of other birth cohorts as follows: All residents of Tromsø aged 42–44 and 60–87 years (*n* = 12,578), a 10% random sample of individuals aged 30–39 years (*n* = 1056), a 40% random sample of individuals aged 43–59 years (*n* = 5787), and finally subjects who attended the second visit of Tromsø 4, if not already included in the three groups above (*n* = 341) [21]. In principle, inviting total birth cohorts is preferable. However, due to economic constraints in the Tromsø 6 survey, a careful consideration of age groups and sample size within the age groups was made on based on scientific evidence and the Tromsø 6 survey protocol.

**Figure 1:**
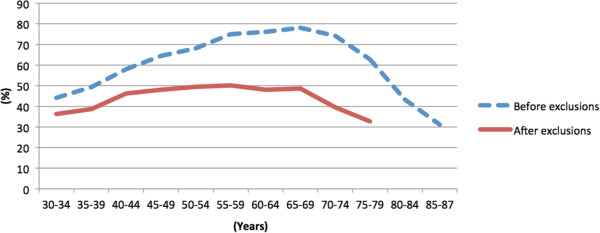
**Attendance rate (%) in the Tromsø 6 survey; first visit by 5-year age group.** The Tromsø Study.

Thus, the Tromsø 6 survey included men and women aged 30–87 years. Of the total participants, 49% were men, and 3% were younger than 35 years. An invitation letter with information about the Tromsø Study and a first questionnaire (Q1) was sent by mail. In total, 12,984 (65.7%) of the 19,762 individuals invited answered Q1 and participated in a brief medical examination, during which various measures were taken (heart rate, blood pressure, height/weight, etc.). Figure 1 shows the participation rate by age group. After the medical examination all participants received a second questionnaire (Q2). A total of 12,440 participants, 62.9% of those invited completed both Q1 and Q2.

### Demographic and lifestyle variables

BMI was calculated from height and weight data collected during the medical examination, and categorized into four groups: <18.5, 18.5-24.9, 25–29.9 and >30 kg/m^2^. Q1 and Q2 are available on the Tromsø Study’s website (http://tromsoundersokelsen.uit.no/tromso/) [22]. Q1 included questions about age, gender, marital status, self-perceived health status, education level, level of physical activity and smoking. The participants were categorized as married if they answered either “married” or “registered partnership”. The question on self-perceived health status had five alternatives: very good, good, neither good nor bad, bad and very bad, which were collapsed into two categories: poor (neither good nor bad, bad and very bad) and good (good and very good) [23]. For descriptive purposes education level was collapsed from five levels into two: <13 years and ≥13 years of education, but in the regression model all five education levels were used. The question on level of physical activity had four alternatives (low, moderate, high, and very high), and smoking status was defined as current, former or never.

Information on chronic MSCs, i.e., suffering from pain and/or stiffness in any of the specified body regions lasting for at least 3 consecutive months during the previous year (hereafter referred to as simply MSCs), was collected from Q2. There was one question for each of the body regions considered: neck/shoulder, arm/hands, upper back, lumbar back, hip/leg/feet and other regions. Participants were asked if they had suffered from pain and/or stiffness in any of these body regions lasting for at least 3 consecutive months during the previous year. The respondents were asked to categorize their symptoms as “no complaints”, “mild complaints” and “severe complaints”. To identify an overall prevalence, we collapsed the answers from all 6 body regions into an overall variable. Those who answered “no complaints” for all 6 body regions were categorized as “no MSCs”. Those who answered either “mild complaints” or “severe complaints” for at least one body region were categorized as “mild MSCs” and “severe MSCs”, respectively. In addition, we computed a variable grouping the respondents by number of body regions reported [8, 11, 24, 25]: 1 region, 2 regions, 3 regions, 4 regions and ≥5 regions.

### Exclusions

**Figure 2:**
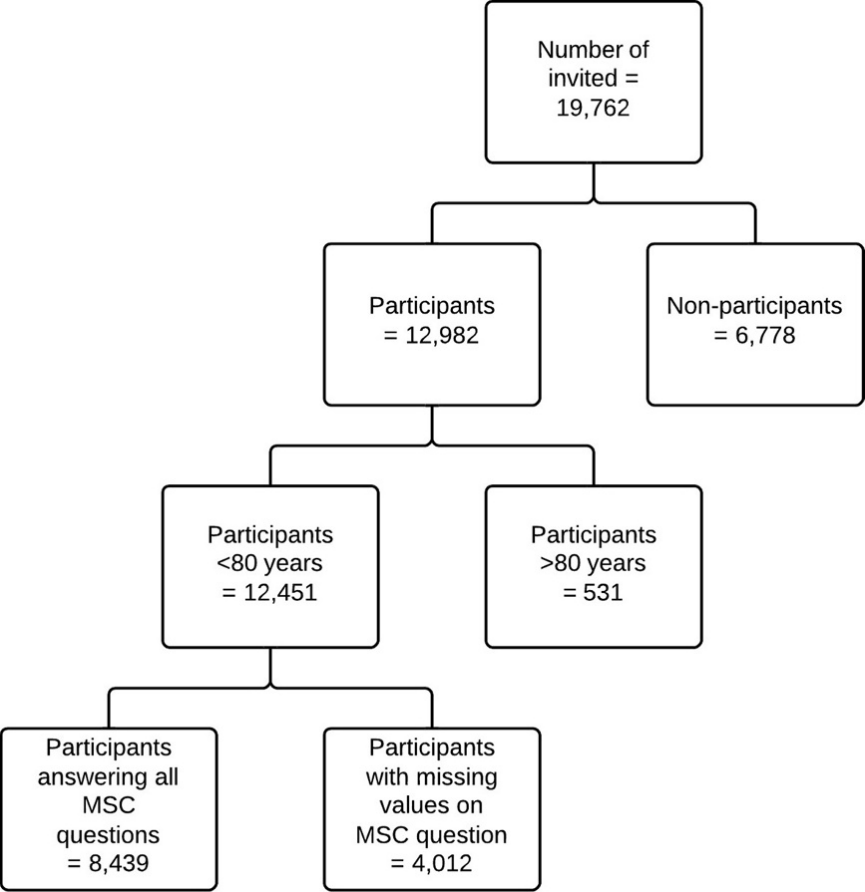
**Flow chart presenting number of subjects invited and those that attended the Tromsø 6 survey in 2007–2008, and those selected for the present analysis.** The Tromsø Study.

Due to low response rates in the age groups 80–84 years and 85–87 years, these age groups were excluded from the analysis. Furthermore, all participants with missing answers to one or more of the questions on MSCs were excluded (Figure 2). A total of 8,439 participants (4,220 women and 4,219 men) remained for the final analyses, providing a real participation rate of 42.7%.

### Statistics

Percentages were used to describe the prevalence of MSCs. Crude prevalence of mild and severe MSCs in women and men were calculated by body region and by age group. Age-adjusted prevalence was calculated using the age distribution of the total population of Tromsø in 2005 (Statistics Norway) as a reference. Differences in demographic and lifestyle characteristics between the three categories of MSCs were compared using ANOVA and the Tukey post-hoc test for continuous variables. The Pearson chi-square test and z-test were used to compare the categorical variables.

We examined the influence of demographic and lifestyle variables on MSCs using univariate and multivariate logistic regression. The analyses were first run for each variable independently, thereafter adjusting for age and gender. Finally, all variables with a significant association in the univariate analyses were included in the multivariate model.

Missing values on demographic and lifestyle variables were: BMI (n = 7), self-perceived health status (n = 47), smoking status (n = 81), marital status (n = 0), education level (n = 72), and physical activity (n = 465). Statistical analyses were performed in SPSS version 19. All tests were two-sided and a *p*-value <0.05 was considered significant.

## Results

The age-adjusted prevalence of mild and severe MSCs was 63.4% for women and 52.9% for men. In women, the age-adjusted prevalence was 44.0% for mild MSCs, and 19.4% for severe MSCs. In men, the corresponding figures were 40.8% and 12.1%. Table 1 shows the prevalence of mild and severe MSCs by 10-year age group and gender. The prevalence of mild MSCs was highest (51.6%) in the oldest age group (70–79 years), which was the only group where men reported more complaints than women (gender ratio: 0.93). The prevalence of severe MSCs was highest in the 50-59-year age group (19.2%), with a gender ratio of 1.55. In the 70-79-year age group, women reported severe MSCs twice as often as men (gender ratio: 2.09).

**Table 1:** **Prevalence of musculoskeletal complaints (MSCs)** ^**i**^ **by 10-year age group in an adult general population in Northern Norway**

Age (years)	Severity of MSCs	Prevalence (%)				
		Total (95% CI)	Women	Men	Gender ratio	***p*** ^*^
30-39	None	49.8 (44.9-54.6)	45.4	55.3	0.82	
n = 408	Mild	36.5 (31.8-41.2)	38.4	34.1	1.13	0.093
	Severe	13.7 (10.4-17.1)	16.2	10.6	1.52	
40-49	None	42.5 (40.6-44.3)	37.8	47.4	0.8	
n = 2,790	Mild	42.4 (40.6-44.2)	44.2	40.5	1.09	<0.001
	Severe	15.1 (13.8-16.5)	18.0	12.1	1.48	
50-59	None	35.4 (33.1-37.7)	31.4	39.7	0.79	
n = 1,697	Mild	45.4 (43.0-47.7)	45.4	45.4	1.00	<0.001
	Severe	19.2 (17.3-21.1)	23.2	15.0	1.55	
60-69	None	35.3 (33.4-37.1)	28.6	41.3	0.69	
n = 2,579	Mild	48.5 (46.6-50.5)	51.1	46.2	1.11	<0.001
	Severe	16.2 (14.7-17.6)	20.3	12.5	1.62	
70-79	None	31.2 (28.3-34.1)	26.7	35.4	0.75	
n = 965	Mild	51.6 (48.5-54.8)	49.8	53.3	0.93	< 0.001
	Severe	17.2 (14.8-19.6)	23.5	11.3	2.09	
Overall^**^	None	41.8 (40.7-42.9)	36.6	47.1	0.77	
n = 8,439	Mild	42.4 (41.4-43.5)	44.0	40.8	1.08	< 0.001
	Severe	15.8 (15.0-16.6)	19.4	12.1	1.60	

^i^Defined as having pain and/or stiffness in muscles and joints at least 3 months during the previous year.

*Pearson chi-square for gender difference.

**Age-adjusted prevalence.

CI: confidence interval.

The Tromsø Study.

Table 2 shows the prevalence of MSCs by body region with regard to severity. The prevalence of MSCs was higher in women than men for all body regions. This gender difference was highly significant, and most pronounced in the group with severe MSCs. The highest prevalence in both men and women was found in the neck/shoulder region, with 34.2% and 8.9% for mild and severe MSCs, respectively. Lumbar back was the second most frequently reported body region for mild MSCs with a prevalence of 28.4%, and the hip/leg/feet region was the most frequently reported for severe MSCs (6.6%), and this distribution was similar in both genders. The largest differences in prevalence by gender were found in other regions and the upper back region, and lowest differences were found in the lumbar back region. All body regions had much lower prevalence of severe MSCs than mild MSCs.

**Table 2:** **Prevalence of musculoskeletal complaints** ^**i**^ **by gender and body region affected in an adult general population in Northern Norway**

		Prevalence (%)				
Body region	N=8,439	Total (95% CI)	Women	Men	Gender ratio	***p*** ^*^
*Neck/shoulder*						
No complaints	4,809	57.0 (55.9-58.0)	50.2	63.7	0.79	
Mild complaints	2,882	34.2 (33.1-35.2)	38.2	30.1	1.27	
Severe complaints	748	8.9 (8.3-9.5)	11.6	6.1	1.89	<0.001
*Arm/hands*						
No complaints	5,936	70.7 (69.7-71.6)	63.8	77.5	0.82	
Mild complaints	2,028	24.0 (23.1-24.9)	28.8	19.2	1.50	
Severe complaints	448	5.3 (4.8-5.8)	7.3	3.3	2.25	<0.001
*Upper back*						
No complaints	6,674	79.1 (78.2-80.0)	72.6	85.6	0.85	
Mild complaints	1,477	17.5 (16.7-18.3)	22.4	12.6	1.78	
Severe complaints	288	3.4 (3.0-3.4)	5.0	1.8	2.84	<0.001
*Lumbar back*						
No complaints	5,573	66.0 (65.0-67.0)	63.2	68.9	0.92	
Mild complaints	2,395	28.4 (27.4-29.3)	29.7	27.0	1.10	
Severe complaints	471	5.6 (5.1-6.1)	7.1	4.1	1.75	<0.001
*Hip/leg/feet*						
No complaints	5,576	66.1 (65.1-57.1)	59.4	72.7	0.82	
Mild complaints	2,309	27.4 (26.4-28.3)	31.8	22.9	1.39	
Severe complaints	555	6.6 (6.0-7.1)	8.7	4.4	1.98	<0.001
*Other regions*						
No complaints	7,722	91.5 (90.9-92.1)	88.9	94.1	0.94	
Mild complaints	613	7.3 (6.7-7.8)	9.2	5.3	1.74	
Severe complaints	104	1.2 (1.0-1.5)	1.9	0.5	3.52	<0.001

^i^Defined as having pain and/or stiffness in muscles and joints at least 3 months during the previous year.

*Pearson chi-square for gender difference.

CI: confidence interval.

The Tromsø Study.

**Figure 3:**
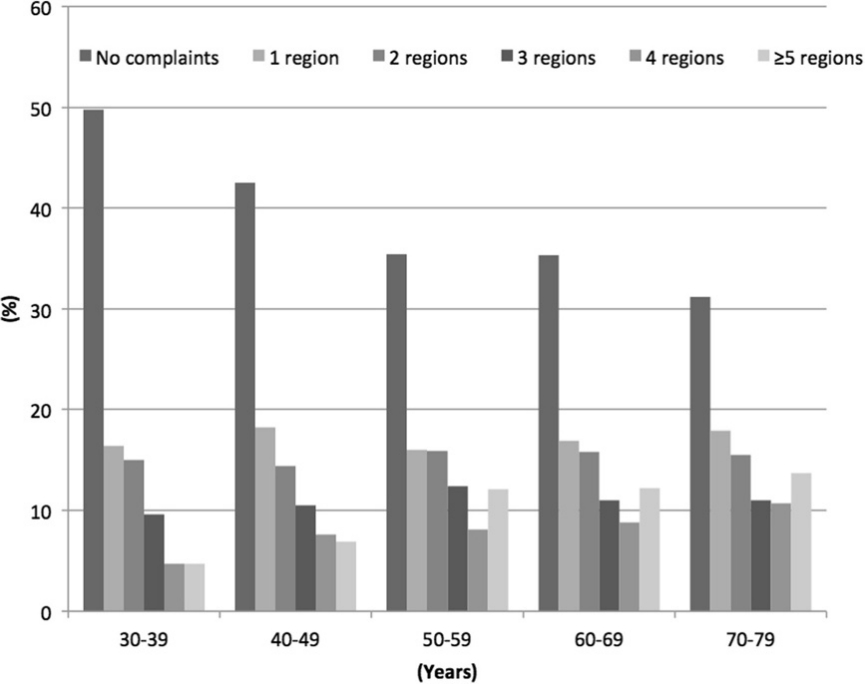
**Prevalence of musculoskeletal complaints** ^***i***^ **by number of body regions affected and 10-year age groups.** The Tromsø Study.^i^ Defined as having pain and/or stiffness in muscles and joints at least 3 months during the previous year.

Prevalence of MSCs by gender and number of body regions affected is shown in Table 3. Overall single-region prevalence of MSCs (either mild or severe) was 17.2% (gender ratio: 0.82) (Table 3). This prevalence decreased with increasing number of body regions affected, except for the group with ≥5 regions, for which the prevalence was higher than in the group with 4 body regions affected (Figure 3). Gender difference became more pronounced with increasing number of regions affected. In the group with ≥5 regions affected, women had a prevalence that was about three times higher than that in men (14.9% vs 5.6%.). More than one-third of the women (35.9%) reported MSCs from three or more regions. Gender differences in multi-region prevalence of MSCs were examined by chi-square testing and found to be highly significant (p < 0.001).

**Table 3:** **Prevalence of musculoskeletal complaints** ^**i**^ **by number of body regions affected in an adult general population in Northern Norway**

		Prevalence (%)				
	N = 8,439	Total (95% CI)	Women	Men	Gender ratio	***p*** ^*^
No complaints	3,200	37.9 (36.9-39.0)	33.0	42.8	0.77	
1 region	1,455	17.2 (16.4-18.0)	15.5	18.9	0.82	
2 regions	1,291	15.3 (14.5-16.1)	14.6	16.0	0.91	
3 regions	931	11.0 (10.4-11.7)	11.8	10.3	1.15	
4 regions	698	8.3 (7.7-8.9)	10.2	6.4	1.59	
≥5 regions	864	10.2 (9.6-10.9)	14.9	5.6	2.66	<0.001

^i^Defined as having pain and/or stiffness in muscles and joints at least 3 months during the previous year.

*Pearson chi-square for gender difference.

CI: confidence interval.

The Tromsø Study.

Table 4 shows the demographic and lifestyle characteristics of the study sample by severity of MSCs. Mean age was significantly different across different severities of MSCs (ANOVA, p < 0.001). The Tukey post-hoc test revealed a significant age difference between women with no MSCs and those with mild MSCs (p < 0.001), and between women with no MSCs and those with severe MSCs (p < 0.001). In men, mean age was only significantly different in the group with no MSCs vs the group with mild MSCs (p < 0.001). Mean BMI was significantly different between groups of MSC severity (ANOVA, p < 0.001), and the Tukey post-hoc test revealed significant differences in mean BMI when comparing no MSCs vs mild MSCs, no MSCs vs severe MSCs, and mild MSCs vs severe MSCs in women (p < 0.001). Whereas in men, there were only significant BMI differences when comparing no MSCs vs severe MSCs (p < 0.001) and mild MSCs vs severe MSCs (p = 0.007). Most of the categorical variables except marital status were significantly different when running the chi-square test (Table 4). Further analyses with the z-test revealed significant differences between all groups of MSCs regarding poor self-perceived health (p < 0.001) and low level of education (p < 0.001) in both women and men. Women and men with severe MSCs (p < 0.001) or mild MSCs (women: p = 0.001, men: p = 0.003) were more likely to be current smokers than those reporting no MSCs. Men with severe MSCs (p = 0.003) or mild MSCs (p < 0.001) were more likely to be former smokers than those reporting no MSCs. In women, former smoking was only significantly different between the groups mild and no MSCs (p < 0.05). Low level of physical activity was significantly different between all groups of MSCs in men (p < 0.05), while in women the difference was not significant in mild vs severe MSCs. Moderate level of physical activity was fairly evenly distributed across the groups, in mild vs no MSCs it was significantly different in both men and women (p < 0.05), and in severe vs no MSCs it was significantly different only in women (p < 0.05). In both women and men high level of physical activity was significantly different in mild vs no MSCs (women: p < 0.001, men: p = 0.002) and in severe vs no MSCs (p < 0.001). Very high levels of physical activity were most commonly reported among those with no MSCs compared to mild (p = 0.003) and severe MSCs (p < 0.05) in both women and men.

**Table 4:** **Distribution of demographic and lifestyle characteristics in women and men divided by severity of musculoskeletal complaints (MSCs)** ^**i**^ **in an adult general population in Northern Norway**

		Women (N = 4,220)				Men (N = 4,219)			
		MSCs				MSCs			
	N = 8,439	None	Mild	Severe		None	Mild	Severe	
		n = 1,393	n = 1,973	n = 854	***p*** ^*^	n = 1,807	n = 1,879	n = 533	***p*** ^*^
Age (years)^**^	54.9	52.5	55.2	55.4	<0.001	54.3	56.5	55.5	<0.001
BMI (kg/m^2^)^***^	26.7	25.3	26.4	27.1	<0.001	27.0	27.3	27.8	<0.001
Self-perceived health status, poor (%)	28.3	10.1^†^	26.1^⊺^	65.1^‡^	<0.001	14.9^†^	32.1^⊺^	58.8^‡^	<0.001
Smoking (%)									
Current	19.6	16.5^†^	21.2^⊺^	29.6^‡^	<0.001	15.7^†^	19.5	23.1^‡^	<0.001
Previous	40.8	35.3^†^	38.6	38.4	<0.001	40.3^†^	46.6	47.6^‡^	<0.001
Marital status (married), (%)	60.4	55.7	58.1	53.7	0.082	64.1	65.6	60.8	0.1
Education level (<13 years), (%)	54.9	44.5^†^	58.8^⊺^	68.7^‡^	<0.001	47.2^†^	58.2^⊺^	68.4^‡^	<0.001
Level of physical activity (%)									
Low	18.8	16.5	17.5^⊺^	20.8^‡^	0.04	17.2^†^	19.9^⊺^	27.6^‡^	<0,001
Moderate	59.6	64.3^†^	69.3	69.8^‡^	0,05	50.0^†^	53.7	50.1	0,07
High	19.6	17.3^†^	12.6^⊺^	8.6^‡^	<0.001	28.9^†^	24.2	20.5^‡^	<0.001
Very high	2.0	1.9^†^	0.7	0.8^‡^	0.002	3.9^†^	2.2	1.8^‡^	0.003

^i^Defined as having pain and/or stiffness in muscles and joints at least 3 months during the previous year.

All values are (%) except for age and BMI (means).

*ANOVA for continuous variables, and Pearson chi-square for categorical variables.

**Tukey post-hoc test: No MSCs vs mild MSCs (p < 0.001) and no MSCs vs severe MSCs (p < 0.001) was significant in women. No MSCs vs mild MSCs (p < 0.001) was significant in men.

***Tukey post-hoc test: No MSCs vs mild MSCs (p < 0.001), no MSCs vs severe MSCs (p < 0.001) and mild MSCs vs severe MSCs (p < 0.001) was significant in women. Tukey post-hoc test: No MSCs vs severe MSCs (p < 0.001) and mild MSCs vs severe MSCs (p = 0.007) was significant in men.

BMI: body mass index.

^†^Z-test: No MSCs vs mild MSCs, p-value <0.05.

^‡^Z-test: No MSCs Severe MSCs, p-value <0.05.

^⊺^Z-test: Mild MSCs vs Severe MSCs, p-value <0.05.

The Tromsø study.

In univariate logistic regression analysis (data not shown) marital status, moderate level of physical activity, and BMI <18.5 kg/m^2^ were not significantly associated with MSCs in either women or men. The other demographic and lifestyle variables showed a positive association with MSCs, except high and very high level of physical activity, which were significantly, but negatively associated. Additionally, we found that the ORs of MSCs decreased from 2.53 (95% CI: 2.22-2.89) in those with the lowest education level to 1.28 (95% CI: 1.12-1.45) in those with the next highest education level, when the highest education level was used as a reference (OR: 1.00). The results from the univariate analyses did not differ when we adjusted for age and gender.

In the multivariate model (Table 5), increasing age (OR: 1.04, 95% CI: 1.01-1.06) and female gender (OR: 1.66, 95% CI: 1.50-1.84) were significantly associated with MSCs. Self-perceived health status was strongly associated with MSCs in both genders combined (OR: 3.73, 95% CI: 3.27-4.24), and in both genders separately (women OR: 4.72, 95%: CI 3.82-5.83; men OR: 3.17, 95%: CI 2.68-3.74). Secondary school as the highest education level was also significantly associated (OR: 1.78, 95% CI: 1.53-2.08) with MSCs. The OR dropped with increasing education level; the next highest education level (<4 years university education) had an OR of 1.19 (95% CI: 1.04-1.37). The association between education level and MSCs showed similar trends in women and men. Current smoking increased the OR for MSCs by 41%, with a larger increase in women than in men (60% vs 25%). Current smoking also showed a stronger association with MSCs than did former smoking (OR: 1.21, 95% CI: 1.09-1.35). The association between former smoking and MSCs did not differ much between women and men. BMI 25–29.9 kg/m^2^ (OR: 1.28, 95% CI: 1.14-1.43) and BMI >30 kg/m^2^ (OR: 1.42, 95% CI: 1.23-1.65) were significantly associated with MSCs. In the gender-stratified multivariate model BMI remained significant only in women. Moderate vs low level of physical activity was significantly and positively associated with MSCs in the study sample, while in the gender-stratified multivariate model this was true only for women. High and very high levels of physical activity were not associated with MSC in either women or men.

**Table 5:** **Demographic and lifestyle characteristics associated with musculoskeletal complaints** ^**i**^ **in 8,439 women and men from an adult general population in Northern Norway in a multivariate regression model**

	OR**(95% CI)		
	Total	Women	Men
**Age (5-year age groups)**	**1.04 (1.01-1.06)**	**1.03 (1.00-1.06)**	**1.04 (1.01-1.07)**
**Gender (women vs men)**	**1.66 (1.50-1.84)**		
**Self-perceived health status (poor vs good)**	**3.73 (3.27-4.24)**	**4.72 (3.82-5.83)**	**3.17 (2.68-3.74)**
**Smoking**			
Never smoker (reference)	1.00	1.00	1.00
Current smoker	**1.41 (1.22-1.62)**	**1.60 (1.30-1.96)**	**1.25 (1.02-1.52)**
Former smoker	**1.21 (1.09-1.35)**	**1.26 (1.08-1.48)**	**1.19 (1.02-1.38)**
**Education level**			
Secondary school	**1.78 (1.53-2.08)**	**1.80 (1.43-2.27)**	**1.76 (1.42-2.18)**
Technical school	**1.59 (1.39-1.83)**	**1.51 (1.23-1.85)**	**1.65 (1.36-2.00)**
High school	**1.25 (1.03-1.51)**	1.22 (0.93-1.61)	1.27 (0.97-1.66)
University <4 years	**1.19 (1.04-1.37)**	1.18 (0.96-1.45)	**1.21 (1.00-1.47)**
University >4 years (reference)	**1.00**	**1.00**	**1.00**
**BMI**			
<18.5 kg/m^2^	0.64 (0.34-1.21)	0.61 (0.30-1.27)	0.75 (0.19-2.89)
18.5-24.9 kg/m^2^ (reference)	1.00	1.00	1.00
25-29.9 kg/m^2^	**1.28 (1.14-1.43)**	**1.51 (1.29-1.77)**	1.06 (0.91-1.24)
>30 kg/m^2^	**1.42 (1.23-1.65)**	**1.76 (1.42-2.19)**	1.14 (0.93-1.39)
**Level of physical activity**			
Low (reference)	**1.00**	**1.00**	**1.00**
Moderate	**1.17 (1.02-1.33)**	**1.37 (1.12-1.67)**	1.02 (0.85-1.22)
High	1.08 (0.92-1.27)	1.23 (0.94-1.59)	0.97 (0.79-1.19)
Very high	0.87 (0.61-1.23)	0.77 (0.40-1.48)	0.83 (0.55-1.26)

^i^Defined as having pain and/or stiffness in muscles and joints at least 3 months during the previous year.

**Multivariate regression analysis (bold text = significant result).

Missing values were: BMI (n = 7), self-perceived health (n = 47), smoking status (n = 81), marital status (n = 0), education level (n = 72), physical activity (n = 465). OR: odds ratio; CI: confidence interval; BMI: body mass index.

The Tromsø Study.

## Discussion

A high prevalence of MSCs was found in this large-scale cross-sectional study. The total crude prevalence of MSCs was 62%, with 45.6% of the participants reporting mild MSCs and 16.4% reporting severe MSCs. Other studies from Norway and Sweden reported a prevalence of MSCs in the general population of 24.4% [10], 34.5% [5] and 50.4% [4]. However, these studies used a screening question with yes/no response alternatives. This is in contrast to the questions in our study, in which the participants were given three alternatives. Thus, a direct comparison is difficult. Some of those who answered mild MSCs may have answered no to a dichotomized yes/no question. If this is the case, it can explain the relatively higher prevalence found in the present study.

To balance this, we chose to present prevalence stratified by the number of body regions affected without taking the severity of MSCs into account. Our finding of decreasing prevalence with increasing number of body regions affected, is in concordance with previous studies [8, 11, 24]. We also showed that men had a higher prevalence than women among those reporting MSCs in one or two body regions, but women reported more MSCs in the groups with more than 3 body regions affected. In addition, among those with ≥5 body regions affected, women had a prevalence that was nearly three times higher than that of men. Similar findings were also reported by Wijnhoven et al. in their 2006 study from the Netherlands [8], and taken together with our study, these findings indicate that women have a high burden of severe MSCs.

The severity of MSCs in our study was dependent on the age distribution – the prevalence of mild MSCs steadily increased with age, whereas the prevalence of severe MSCs peaked at around 50–59 years of age. The steady increase in mild MSCs could reflect an physiological effect related to the aging process, and the decline of severe MSCs after 60 years of age may reflect reduced mental and physical stress after retirement [26] or increased mortality in this group [27].

Like other studies, we found the highest prevalence of MSCs in the neck/shoulder and lumbar back regions. However, a direct comparison of the prevalence that we found with that in other studies is difficult because the present study put the neck and shoulder together in one body region, while others separated these into two body regions [4, 8], or combined them with different body regions [11, 12, 14]. Hasvold found a prevalence of daily neck/shoulder pain in a Northern Norwegian population (1989/1990) of 7.8% for men and 12.5% for women [20]. We found nearly the same prevalence as that we found for severe MSCs in the neck/shoulder region, despite some differences in the questionnaire, which indicates that the prevalence of MSCs in Northern Norway has been quite stable.

Most of the previous studies evaluating characteristics associated with MSCs did not stratify their regression analyses by gender. Thus, only the gender-independent ORs can be compared. Some studies reported an increasing OR for MSCs with increasing age [4, 5, 9], which is in concordance with our findings, while others found age to be either protective or not related [6, 10]. The positive association between age and MSCs might be explained by an accumulation of associated demographic and lifestyle variables over time, the effects of which increases with age.

Some [4, 6, 8–10], but not all [5, 14] studies have shown that female gender is associated with reporting MSCs. We demonstrated that female gender increased the OR of having MSCs by 66%. Women also reported more multi-region and severe MSCs than men. Hagen et al. [4] found an association between current smoking and MSCs. We were also able to show this association in our model stratified by gender, just as Palmer et al. [18] reported. Additionally, we found former smoking to be associated with MSCs, but this relationship was weaker than for current smoking. There was a larger gender difference in the association between current smoking and MSCs than between former smoking and MSCs. This suggests that quitting smoking could greatly reduce the burden of MSCs, and that women would benefit more from this reduction than men.

When dichotomizing the BMI into 4 levels, we found obesity (BMI >30 kg/m^2^) to be positively associated with MSCs, which is in concordance with the report by Hagen et al. [4]. Additionally, we found that underweight was not associated with MSCs in either direction. Our findings on the association between BMI and MSCs could suggest that body weight exerts a mechanical stress on the musculoskeletal system that gives rise to development of MSCs. However, it is strange that this should only be true for women. Since we cannot infer any causal association due to our cross-sectional design, it is indeed possible that MSCs contribute to obesity.

Some studies have reported that low education level is not associated with MSCs [5, 6], in contrast to reports by Rustøen et al. [10] and Hagen et al. [28]. In our univariate analyses we found an inverse relationship between education level and MSCs, which persisted in the multivariate model and was almost identical for men and women. From a clinical perspective, this might be of interest. Indeed, paying more attention to those with the lowest education level could potentially have positive consequences in preventing MSCs. Marital status was not associated with MSCs in any of our analyses, including the multivariate analyses, which is in concordance with Rustøen et al. [10] and Bassols et al. [6]. Therefore, in the final multivariate regression model we did not include marital status. In addition to education level, marital status was the only associated factor where the strong female association with MSCs was not present.

Self-perceived poor health status was strongly associated with MSC in the multivariate analysis and, interestingly, was on a similar level as that reported by Hagen et al. [4]. This association may not be so interesting when discussing the potential risk factors of MSCs, because there is a substantial chance that a high burden of MSCs leads to poor self-perceived health status, and not vice-versa. However, if that is true, this association tells us that MSCs have a major negative influence on a person’s health and well-being, which may have crucial health effects [27].

In our univariate analyses, increasing level of physical activity was negatively associated with MSCs in both women and men. After adjusting for other covariates in our multivariate model, the relationship between physical activity and MSCs was attenuated. However, a moderate level of physical activity was positively associated with MSCs in women. Holth et al. reported that exercise had a preventive effect on MSCs in a prospective study [19], but we were not able to show such a clear association in our study. Our contradictory findings on physical activity might be a result of confounding bias or the fact that this is a cross-sectional study and that the outcome variable (MSCs) is influencing the level of physical activity unevenly among our participants.

The finding that many of the association with demographic and lifestyle variables, such as current smoking and BMI, was stronger in women, is an interesting one and might give us a hint as to the important aspects of the pathophysiology of MSCs. Thus, this gender difference warrants further research.

### Strengths and limitations

This analysis included a large number of women and men with a wide age range from a large population-based study. The overall representativeness of the population of the Tromsø Study is described elsewhere [21]. When stratified by age group, the attendance rate tended to be normally distributed; the youngest and oldest participants had the lowest attendance rate (Figure 1). It is possible that respondents in the youngest age group had a higher burden of disease than those who do not respond, and therefore might have been more engaged in research on health issues. If this is true, our study might overestimate the burden of MSCs in the youngest part of the population. However, we included the age group 30–39 years, despite low attendance, because MSCs are reported to be increasing in the young Norwegian population [4]. Inclusion of participants in the age group 20–29 years would be a great contribution to the field, and should be included in future studies. The generalizability of the present findings should be limited to those 30–80 years old.

Many participants were excluded from the analysis because of missing answers to relevant questions. These participants might selectively answer only questions about complaints they had and left the rest of questions unanswered. In sensitivity analysis where all missing values were coded as negative answers, the prevalence was slightly higher (mild MSCs: 48.2%, severe MSCs: 18.2%), but the regression results remained unchanged.

More excluded participants were female (59.5% vs 50%), older (59.8 vs 54.9 years), had poor self-reported health status (43.7% vs 28.3%), and less had a high education level (26.9% vs 45.1%) compared to those included. This could have led to the underestimation of the associations found in the regression analyses.

In future studies it is essential that identical phrasing of the questions be used, so that comparison and incidence analyses can be done. However, one should consider asking for only a yes/no response for these variables. Indeed, the questionnaires in the Tromsø study are already wide-ranging, and a simplification of the variables would make the participants’ task easier.

## Conclusion

Chronic MSCs are prevalent in this Northern Norwegian population, and self-perceived poor health was strongly related to MSCs. Women had a higher burden of MSCs than men. The prevalence decreased when the severity of complaints increased. The lifestyle factors associated with MSCs were stronger in women than in men. This gender difference needs to be further investigated.
